# Dynamics, Patterns and Causes of Fires in Northwestern Amazonia

**DOI:** 10.1371/journal.pone.0035288

**Published:** 2012-04-16

**Authors:** Dolors Armenteras, Javier Retana

**Affiliations:** 1 Landscape Ecology and Ecosystem Modelling Laboratory, Department of Biology, Sciences Faculty, Colombia National University, Bogotá, Colombia; 2 CREAF i Unitat d'Ecología, Universitat Autònoma de Barcelona, Barcelona, Spain; DOE Pacific Northwest National Laboratory, United States of America

## Abstract

According to recent studies, two widespread droughts occurred in the Amazon basin, one during 2005 and one during 2010. The drought increased the prevalence of climate-driven fires over most of the basin. Given the importance of human-atmosphere-vegetation interactions in tropical rainforests, these events have generated concerns over the vulnerability of this area to climate change. This paper focuses on one of the wettest areas of the basin, Northwestern Amazonia, where the interactions between the climate and fires are much weaker and where little is known about the anthropogenic drivers of fires. We have assessed the response of fires to climate over a ten-year period, and analysed the socio-economic and demographic determinants of fire occurrence. The patterns of fires and climate and their linkages in Northwestern Amazonia differ from the enhanced fire response to climate variation observed in the rest of Amazonia. The highest number of recorded fires in Northwestern Amazonia occurred in 2004 and 2007, and this did not coincide with the periods of extreme drought experienced in Amazonia in 2005 and 2010. Rather, during those years, Northwestern Amazonia experienced a relatively small numbers of fire hotspots. We have shown that fire occurrence correlated well with deforestation and was determined by anthropogenic drivers, mainly small-scale agriculture, cattle ranching (i.e., pastures) and active agricultural frontiers (including illegal crops). Thus, the particular climatic conditions for air convergence and rainfall created by proximity to the Andes, coupled with the presence of one of the most active colonisation fronts in the region, make this region differently affected by the general drought-induced fire patterns experienced by the rest of the Amazon. Moreover, the results suggest that, even in this wet region, humans are able to modify the frequency of fires and impact these historically well preserved forests.

## Introduction

Long-term decreases in annual rainfall, recent drought episodes related to the Amazon basin and the sensitivity of the Amazon rainforest to these drying trends are being discussed extensively [1], [2], [3], [4]. Both the El Niño Southern Oscillation (ENSO) and other sea-surface temperature events, such as the Atlantic Multidecadal Oscillation, have been associated with drought events in Amazonia [3], and evidence has even been found of extensive droughts that were associated with paleo-ENSO events in south-western Amazonia [5]. The rising temperatures and altered rainfall resulting from these drying trends may decrease the levels of humidity in the region, with the potential to transform the rainforest into savannah vegetation and pastures [2].

Fires for land clearing and management are one of the major sources of carbon emissions from Amazonia and play an important role in the vegetation-atmosphere interactions and the hydrology of the area [6]. The extreme drought conditions described above increase the amount of area burned and the frequency of fires, and they produce longer fire seasons [7]; droughts co-occur with peaks of fire activity [4]. The reason for this relationship is that severe droughts increase the flammability of the forests because hydrological factors, including the amount of floodplain area, are significantly affected by drought [3]. This process affects the sensitivity of the forests to dry conditions, increases leaf litter dryness and produces tinder for wildfires [1], [8]. In the Amazon basin, recent fires are also typically associated with drought periods [3], [5], [9]. Indeed, a reported increase in forest fires in the Amazonian Basin has been associated with extreme climatic events, such as the El Niño Southern Oscillation (ENSO) [10], [11] or the warm tropical North Atlantic oscillation [5]. Thus, the potentially critical role of fire has been apparent during the recent droughts in Amazonia, with extensive fires spreading from agricultural zones into flammable forests during the droughts of 1997, 1998, 2005 and 2007 [10], [12].

Humans also play a major role in determining fire patterns in the earth system [13]. The use of fire for land clearing and management is one of the major threats to neotropical forests and particularly in the Amazon [14]. Current economic and demographic growth has favored increasing demands for agricultural land and timber products from tropical forests [15] and thus has promoted management practices that increase the risk of forest fires. The land use conversion to crops and thus the expansion of the agricultural frontier has been reported as cause of increased deforestation and fire in the Amazon [6], [16]. In some cases this expansion has been strongly influenced by illicit crops, especially in remote areas,frequently related to armed conflict and population displacement [17], [18]. Fire frequency is also higher in forests under logging and forests that are being cleared for cattle ranching and converted to pastures [19]. Further fire in the Amazon occurs more frequently in fragmented forests, near roads and close to town centres [19], [20].

In 2005, below-normal precipitation occurred across most of South America, except in the western and south-western parts of the continent. During the same year, large areas of the Amazon basin experienced one of the most intense droughts recorded in the past 100 years, with Southwestern Amazonia as its epicentre [3], [10], [21], [22]. Current reports indicate that the 2010 Amazonian drought is likely to have been less severe but more spatially extensive than the 2005 drought [4]. However, these drought periods are not homogeneous but vary spatially and temporally throughout the Amazon basin [3]. Typically, the dry season lasts 4–5 months (with <100 mm of rainfall per month) in much of the eastern and southern portions of the basin [22], [23], but Northwestern Amazonia is the least likely part of the basin to experience major drought. The 2005 drought was also characterised by extensive fires in the South-western region, and the cumulative number of fires in Amazonia increased by 33% in 2005 relative to that recorded in previous years [3], [24].

Studies on drought periods and their linkages with fire patterns have evaluated the whole Amazon and focused on parts of the basin where droughts have been most extensive [6], [10], [21]. However, the analysis of active fires previously observed in Northwestern Amazonia suggests that the patterns of fire and climate in this part of the basin do not reflect the dynamics of the rest of the basin [25]. Northwestern Amazonia is one of the wettest tropical rainforest regions, with *ca.* 3000 mm of rain per year. Its dry season is shorter and less intense than the dry season in Southwest, Southeast or Central Amazonia [9]. Moreover, different sources indicate that the drought in 2005 was concentrated in western and southern Amazonia and did not have comparably severe effects in northern or eastern Amazonia [5]. On the other hand, in this area the Andes create conditions for air convergence and rainfall that differ from those found in the rest of the basin [26], [27]. Moreover, this region is of a particular nature because it is one of the most active colonization fronts in the region through the expansion of the agricultural frontier and the presence of illicit crops, logging activities and pastures [28], a cluster of factors that determines the pattern of fires in most cases [25].

This study compares the spatio-temporal variability of rainfall with that of fires over the past decade in Colombian Amazonia. We also analyse the role of potential drivers of fire occurrence (i.e., deforestation, population, pastures, crops and illicit crops) on the distribution of fires in the region. Given the statements described above, our main hypothesis is that fire incidence will have stronger relationships with human drivers than the fire response to climate variation observed in other parts of the Amazon

## Methods

**Figure 1:**
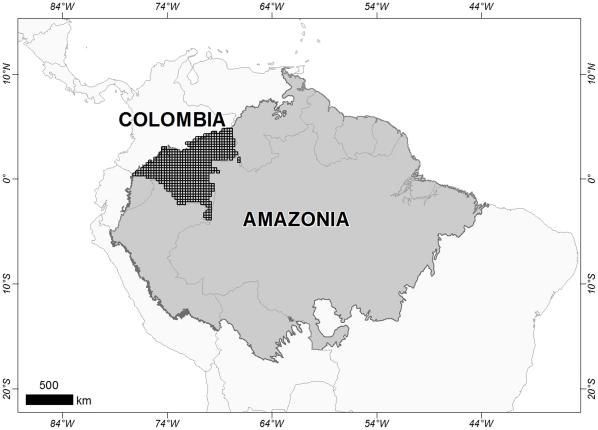
Map of the study area that depicts the limits of Amazonia and the Colombian part of the basin covered in this study.

The Amazon basin has a total area of 6.3 million km^2^, with 5 million km^2^ in Brazil and the remaining area divided among Bolivia, Colombia, Ecuador, Peru and Venezuela. The study area (Fig. 1) corresponds to the Northwestern part of the Amazon, in particular the Colombian Amazon region. This region includes 7% of the entire Amazon Basin, and 40% of it is south of the equator. The population density of the region is still low, but the area is a very active colonisation front [25], [29]. The region is highly diverse, owing mainly to differences in geology and geomorphology that produce environments with a great variety of drainage systems and soil qualities. These conditions produce very important differences in ecosystem composition and structure. Several different types of vegetation occur in the region. Lowland forests (<600 m) constitute the first type and can be classified as either riparian or periodically flooded forest (*Várzea* or *Bosques temporalmente inundables*) or moist or permanently flooded forest (*Igapó* or *Bosques Inundables*). Upland forests are similarly differentiated into riparian upland forest (*Campinarana, Bosques de Tierra Firme or Bosques de Colinas*) and montane upland forest (*Piedemonte, Sierra*). The western Amazon is also home to many isolated summits, which support tepui vegetation and montane savannas characterised by high levels of endemism (Tepuis, Pantepui). Various types of aquatic and swamp vegetation complexes are found along the major rivers.

Rainfall data were obtained from the Tropical Rainfall Measuring Mission dataset (TRMM) from January 1998 to February 2011 at 0.25° ( = 25 km) spatial resolution. A monthly cumulative rainfall dataset was derived for Colombia from the original daily dataset and from the TRMM V6 dataset [30], [31]. The cumulative rainfall per three-month period was calculated by summing the values for December (year *i*-1), January and February (year *i*) (hereon referred to as DJF of year *i*). This trimester was chosen because it is the period during which the highest number of fires occurs in the region and generally corresponds with the dry season [25]. Three-month rainfall standardised anomalies in each year *i* (TRMM_anomaly, i_) were also quantified per pixel as the difference between the precipitation of year *i* (TRMM_i_) and the 2001–2010 mean (TRMM_2001–2010_) normalised by the standard deviation (σ_2001–2010_) (eq.1; see [10], [25]).
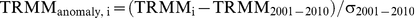
(1)where positive values indicate a wetter DJF period in year *i* than the average rainfall for the 2001–2010 DJF period and negative values indicate a drier-than-average DJF period in year *i*.

We used the fire-hotspots series product MODIS, which is included in the Collection 5.1 temporal thermal analysis active-fire dataset [32]. We downloaded the daily dataset ranging from December 2000 to December 2010; this dataset is available at FIRMS (Fire Information for Resource Management System: Archiving and Distributing MODIS Active Fire Data, Collection 5.1 [32]). Active fire detection was based on detecting the thermal signature of fires using a contextual algorithm [33]. We obtained the number of hotspots in each of the 580 0.25° cells considered in the study area. No specific permits were required to access the datasets for these locations.

Deforestation data from 2000 and 2005 were obtained from the official project “Scientific and institutional capacity building to support Reducing Emissions from Deforestation and Degradation (REDD) projects in Colombia” [34]. Information concerning pasture and crop cover was derived from the official cartography of Colombia's continental, coastal and marine ecosystems, which originally used Landsat 2000–2002 images [35] that were available on a 1∶500,000 scale. One of the limitations of the study was that we could only use information matching the start of the study period, but the data described above are the only information available for our study period that covered the whole study area. Information regarding the growth of illicit crops in the region was obtained from the global illicit crop-monitoring program of the United Nations Office on Drugs and Crime [36], which is available in Colombia through the Integrated System for Illicit Crops Monitoring project or SIMCI (Sistema Integral de Monitoreo de Cultivos Ilícitos). This system has been consistently monitoring illicit crops in the region since 2000 and provided us access to coca survey data for the study area. We used the GIS package ArcGIS (ESRI) to conduct all digital spatial analyses. Finally, we included data on urban population obtained from the 1985, 1993 and 2005 population censuses [37].

The analyses of the variations of numbers of fire hotspots and climatic variables among the different years of study (from 2001 to 2010) were performed with repeated measures ANOVA tests, with the year serving as the repeated measures variable. In these analyses, the sampling units in which we estimated rainfall and fire values were the 580 0.25° cells. Statistical analyses were carried out using STATISTICA 6.0.

The relationships between climate and fire variables were analysed using non-parametric Spearman correlations with two different datasets. First, we analysed the relationship between the number of hotspots, precipitation and anomalies in precipitation in each of the ten years of the study using the values obtained in the 580 cells sampled each year. We also carried out the correlation analyses of fire and climate variables using the mean values per year of the cell values. In this case, the sampling units were the ten years of the study period (from 2001 to 2010).

We also carried out non-parametric Spearman correlations to determine the relationship between the number of hotspots per cell and the following five explanatory variables: % deforestation, % pastures, % crops, surface of coca crops and population. The 580 0.25° cells were again used as the sampling size in these analyses.

## Results

**Figure 2:**
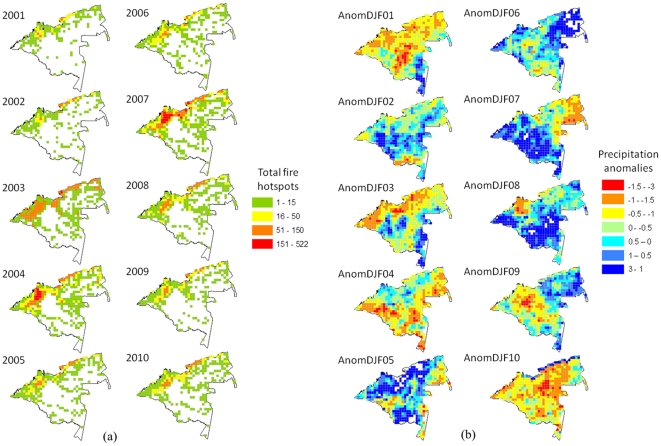
Spatial distribution of the (a) number of fire hotspots and (b) rainfall anomalies in the period including December of the previous year and January and February of the current year (DJF) from 2001 to 2010 in the entire study area.

Fig. 2 illustrates the spatial distribution of hotspots (a) and precipitation anomalies (b) in the study area for the DJF of each year analysed. In general, these maps show areas with high densities of fires and higher values to the west, towards the Andes. The correlation between the numbers of hotspots per cell in different years was highly significant (correlation values between years ranged between 0.69–0.92), confirming the high recurrence of fires. Slightly different results were found for the precipitation anomalies. A distinct pattern was found for each year. The years 2001, 2003, 2004 and 2010 clearly had more negative precipitation anomalies (i.e., were drier) than 2002, 2005, 2006, 2007, 2008 or 2009. In 2004, the negative precipitation anomalies occurred around the central and the western parts of the study area, whereas in 2010, the negative precipitation anomalies occurred around the northeast of the study area. In 2001, negative anomalies were registered in the central and the western parts of the study area, but in 2003, the drier areas were generally found in the lowland Andes.

**Figure 3:**
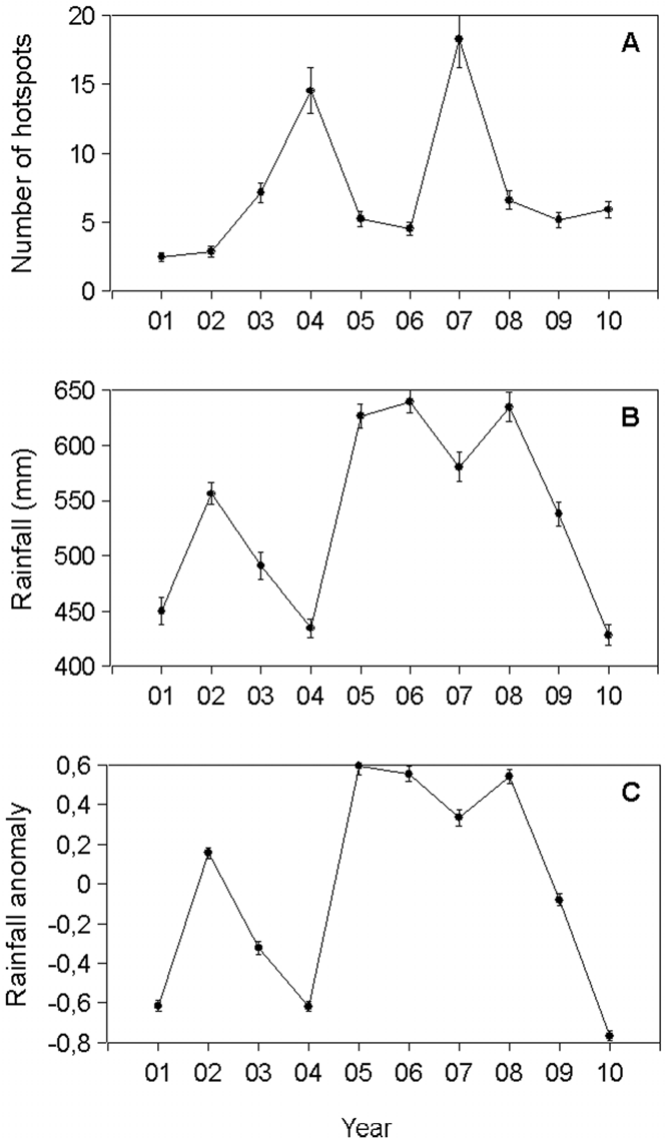
Mean values (±standard error of the mean) of the (a) number of fire hotspots (HP) in the period including December of the previous year and January and February of the current year (DJF); (b) accumulated rainfall in the DJF period (mm); and (c) precipitation anomalies in the DJF period between 2001 and 2010 (without units).

The fire hotspots in the study area exhibited strong variability among the years of study (repeated measures ANOVA, F_9,5211_ = 57.5, p<0.001). The highest number of fire hotspots detected was 18.3±2.1 hotspots per cell (mean±standard error, N = 580 cells) in 2007, followed by 14.5±1.7 in 2004. These two years had exceptionally high values compared with the rest of the period analysed (Fig. 3a).

The temporal patterns of precipitation analysed for the DJF periods between 2001 and 2010 also indicated large interannual variability (F_9,5211_ = 197.5, p<0.001, Figure 3b). The years 2005, 2006 and 2008 were the wettest years, with 626±11, 639±10 and 634±13 mm of rainfall accumulated in the DJF trimester, respectively. At the other extreme, the years 2001, 2004 and 2010 had only 450±12, 434±8 and 428±9 mm of rainfall, respectively. The same pattern was observed for the rainfall anomalies (F_9,5211_ = 232.9, p<0.001; Figure 3c). In all cases, the anomalies reported were less than one standard deviation, indicating low deviations from the mean. The most positive anomalies indicated wetter-than-average periods in 2006 (0.55±0.37), 2008 (0.54±0.036) and 2005 (0.59±0.04). In contrast, the driest periods with negative anomalies were found in 2010 (−0.76±0.02), 2004 (−0.61±0.02) and 2001 (−0.61±0.02).

The spatial relationships of the different climate and fire variables in the different years did not show similar patterns. The relationship between the number of fire hotspots per cell and the accumulated rainfall per cell was negative in all ten years considered (Spearman correlation; Rho between −0.15 and −0.55; p<0.001 and n = 580 in all cases), indicating that the number of fire hotspots was higher in cells with lower precipitation. The relationship between rainfall anomalies and hotspots was generally not consistent among the years of study. Two years showed a positive relationship (2004: Rho = 0.14; 2005: Rho = 0.27; p<0.05 and n = 580 in both cases), and six years showed a negative relationship (2001: Rho = −0.23; 2002: Rho = −0.17; 2003: Rho = −0.39; 2007: Rho = −0.11; 2008: Rho = −0.20; 2009: Rho = −0.16; p<0.01, n = 580); in 2006 and 2010, the relationship was not significant. In 9 out of 10 years, the relationship between the accumulated rainfall and the rainfall anomaly was positive (Rho between 0.33 and 0.79; p<0.01 and n = 580 in all cases), indicating that the anomalies in rainfall were higher in wet cells than in dry cells.

When considering mean annual values of fire and climate variables from all cells, no correlation was found between the number of fire hotspots and either the rainfall or the precipitation anomalies (p>0.80 in all cases, n = 10 years).

**Figure 4:**
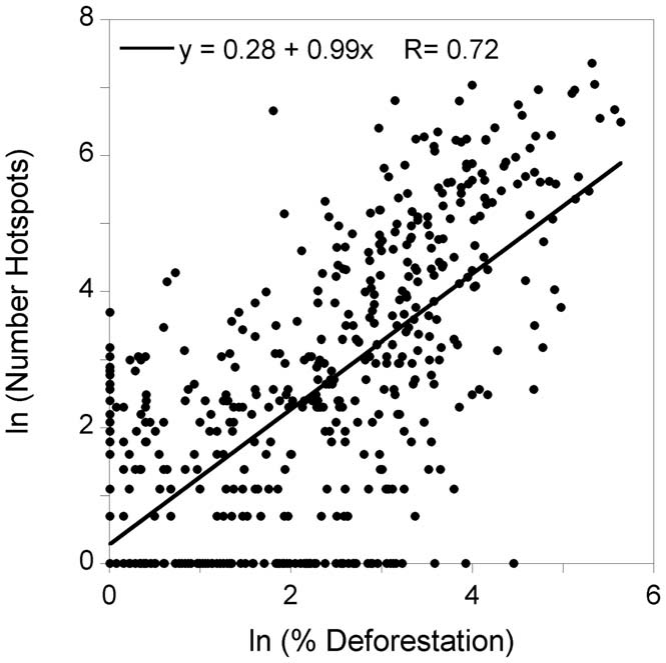
Relationship between the number of hotpots per cel (log-transformed) and % deforestation (log-transformed). N = 580 cells.

There was strong relationship between the number of hotspots per cell and % deforestation (Rho = 0.72, p<0.001, N = 580; Figure 4). The other four human-related variables considered showed a high correlation with % deforestation (% pastures: Rho = 0.64, surface of coca crops: Rho = 0.44, %, population: Rho = 0.27, crops: Rho = 0.18; p<0.001, N = 580 in all four cases), confirming their role on forest loss. The consequence is that the relationship between the number of hotspots per cell and these four explanatory variables was also highly significant and positive: % pastures (Rho = 0.76, p<0.001, N = 580), surface of coca crops (Rho = 0.64, p<0.001, N = 580), population (Rho = 0.25, p<0.001, N = 580) and % crops (Rho = 0.17, p<0.001, N = 580).

## Discussion

The spatio-temporal variability in rainfall and fire patterns differs throughout the Amazon basin. Northwestern Amazonia is one of the wettest tropical rainforest regions and does not yet exhibit the strong effects of drought reported for other areas of Amazonia. The Andes, specifically their eastern slope, create positive conditions for air convergence and rainfall in Colombian Amazonia [26]. Our results confirm the findings of Malhi et al. [21] and others [5], [10], [26] that Northwestern Amazonia has a weaker dry season than southwest, southeast or central Amazonia [9], with very weak (less than 1 standard deviation) anomalies reported for the ten years considered.

The 2005 drought was reported to be more severe in the Southwestern Amazon than in the Central and Eastern region [5]. Precipitation anomalies for the 2005 DJF period in Northwestern Amazon were unlike the drought conditions experienced elsewhere in the basin. On the contrary, the DJF period in this area was quite wet. The 2004 and 2007 years are associated to the ENSO phenomenon, as it has been previously reported for Colombia [25], [27]. Precipitation anomalies for these two years were two of the lowest for the period considered (Fig. 3). In 2010, the DJF period was dryer, with more precipitation anomalies. Nevertheless, neither an ENSO dry season as 2004 nor the 2010 DJF period registered rainfall below 400 mm. Dought expansion should be monitored for this region in the future because precipitation anomalies are rarely reported to occur in the north-western part of the Amazon, as this area has been the least affected by historical climate variability [2].

The results of this study reveal that, although there is a relationship between the number of fire hotspots and the accumulated rainfall, no strong interaction exists between climatic events and fire incidence, the latter of which is relatively low when compared to other regions in the Amazon where fire is a major problem and may reach more than 25 counts per 0.25° cell [10]. These results confirm that drought-enhanced fires do not occur equally over the entire Amazon and that Northwestern Amazonia has a pattern particular to its geography. In our analysis (see also [6]), Northwestern Amazonia experienced the highest number of fires in 2004 and 2007, and this did not correspond to the years of highest fire incidence and extreme drought recorded in other parts of Amazonia. These two years were El Niño years and thus ENSO events might be affecting the dynamics of fire more strongly in this part of the basin. In fact during the extreme Amazonian droughts of 2005 and 2010, Northwestern Amazonia exhibited a relatively low number of hotspots and 2005 was one of the wettest periods. Furthermore, there were a large number of fires in 2004, one of the driest periods recorded during our study period but not in other parts of the basin. This finding indicates that fires are more common in Northwestern Amazonia during periods of lower rainfall associated to El Niño events (although we cannot call them strictly drought periods), which is contrary to the association to increased SST in the Atlantic Ocean trends in other subregions of the Amazon (e.g., [3], [10]).

There was no relationship between climate and fire occurrence at the annual level, and this was in agreement with the results obtained by Armenteras *et al.* [25]. However, the spatial analysis at the cell level revealed a negative relationship between the number of fire hotspots and rainfall in all ten years tested. This result suggests that fire activity is more severe in the driest areas. In addition, fires show a clear recurrence over the years. The same areas show the greatest fire activity in multiple years (Fig. 3a), probably as a consequence of the impact of social drivers of fire (see below). However, the cell-level relationship between the number of fires and the rainfall anomaly showed contradictory patterns among the years of study. This result confirmed the lack of a clear interaction between fires and climate in the region.

All these results underscore the general notion presented in previous studies [7], [25], [38] that the pattern of forest loss and fire occurrence in Northwestern Amazonia is not fully limited by the extent of the dry season but rather is influenced much more strongly by socio-economic factors [7]. The process of immigration began recently (in the 1960s) in Amazonia and this region contains currently active colonization and deforestation fronts [28], [29]. Many of the active fires detected occurred in areas of transition between the submontane and lowland Andes and Amazonia, where the expansion of the agricultural frontier via the river network is evident [28]. This study provides evidence of fire-driven deforestation, land-use change and colonisation fronts occurring in this part of the Amazon basin.

We have shown that fire occurrence correlates well with anthropogenic drivers. There was a high correlation between fire occurrence and deforestation, indicating that there was a clear association between the two phenomena (see also [4]). Moreover, the other human-related variables considered, which are closely correlated with deforestation, also determined the pattern of fires. In fact, small-scale agriculture, cattle ranching (i.e., pastures) and active agricultural frontiers (including illegal crops) are evident in the region, and fire is the primary tool used to clear forest and maintain areas of pastures and farmlands. Pasture conversion is the primary land use change occurring while human-related fires for the purpose of land clearing and for soil preparation are also common in the area. Recurring fire activity in the process of conversion to pastures has been reported for other parts of the Amazon [8] as one of the strongest land use changes. The conversion of forest to pastures has been recognised as a continuous process across Latin America [19]. Besides the fact that land occupation in the region is based on the clearing of primary forest on pastures, the expansion and intensification of agriculture does not yet exist. This might be partly due to the illicit crop economy, which is a temporary exploitation of a crop strongly linked to armed and illegal groups that frequently leads to land abandonment, resulting in an increase in secondary transformed ecosystems and fire risk.

There is still much to understand regarding the impacts of fires, the exact mechanisms by which fire operates in this part of the Amazon and how activities such as cattle ranching or the selective logging of forests may increase the risks of the fire dispersal into the rainforest. Malhi *et al.* [2] suggest that “rain forests may become seasonally flammable in dry years but without anthropogenic ignition sources fire is a rare occurrence”. Thus, obtaining a better understanding of the interaction dynamics between human activities, fire, and climate (as in [20], [38] becomes a priority for this area. The results obtained in this study suggest that, even in wet regions such as Northwestern Amazon, where fires were historically rare, humans are able to modify the frequency of fires and impact these well preserved forests (as predicted by [14]. Ultimately (governmental) initiatives to use alternative land management techniques in the colonisation front are urgently required, together with the establishment of consistent and strong fire and land use monitoring programmes and the reinforcement of a sustainable development plan for the region.
